# Short- and Long-Term Antidepressant Clinical Trials for Major Depressive Disorder in Youth: Findings and Concerns

**DOI:** 10.3389/fpsyt.2019.00705

**Published:** 2019-10-11

**Authors:** Daniel J. Safer, Julie Magno Zito

**Affiliations:** ^1^Department of Psychiatry, Johns Hopkins University School of Medicine, Baltimore, MD, United States; ^2^Department of Pediatrics, Johns Hopkins University School of Medicine, Baltimore, MD, United States; ^3^Department of Pharmaceutical Health Services Research, University of Maryland Baltimore, Baltimore, MD, United States; ^4^Department of Psychiatry, University of Maryland, Baltimore, Baltimore, MD, United States

**Keywords:** discontinuation trials, antidepressants, maintenance, relapse, attrition, major depressive disorder, adolescent, efficacy

## Abstract

The diagnosis of major depressive disorder (MDD) in U.S. youth is increasing as is the rate of antidepressant medication (ADM) treatment for the disorder. Fluoxetine and escitalopram are FDA approved for the short term and maintenance treatment of MDD in youth. Placebo-controlled short-term ADM trials represent the basis for Food and Drug Administration (FDA) approval. Meta-analyses in 2007 and 2016 revealed that short-term ADM treatment of youth diagnosed with MDD resulted in no meaningful benefit for children and only marginal benefit for adolescents. Placebo substitution trials of ADM short-term responders represent the basis for FDA approval of ADM maintenance treatment. These ADM placebo substitution maintenance trials for youth with MDD are characterized by high dropout rates, a rapid withdrawal that often can follow the switch to placebo, and relapse rates that are not dissimilar from those in the natural course of the disorder. Without the evidence from problematic ADM placebo substitution trials, there is no acceptable support for the inclusion of ADM in maintenance treatment for MDD in youth.

## Antidepressant Treatment for Major Depressive Disorder in Youth

Antidepressant treatment (ADM) is the second most common psychotropic therapy for U.S. youth (1), and in recent years its treatment prevalence is increasing (2, 3). Fluoxetine is FDA approved for youth age 8 and up diagnosed with MDD for acute and maintenance treatment (4). Also, escitalopram is FDA approved for the acute and maintenance treatment of MDD for adolescents (5). Support for the short-term use of antidepressants is based on 8-12 week randomized, placebo-controlled trials. Maintenance ADM treatment has its FDA support based on double-blind, placebo substitution trials of short-term ADM responders. Such trials are also referred to as drug discontinuation and relapse prevention trials. In these trials, one-half of antidepressant medication clinical trial responders are randomly selected to be switched abruptly from their ADM to placebo immediately after entering the trial.

This review initially presents a brief summary of the evidence from ADM short-term trials for youth diagnosed with MDD. The data combines findings from SSRI with SNRI ADM subclasses–since they have similar trial outcomes (6) —and does not specifically deal with tricyclic ADMs trials. The emphasis in ADM trial outcome is on treatment response (a 50% or more symptom reduction from baseline, or a major improvement in a researcher assessed treatment judgment—using the CGI-I), not remission. The term continuation is used interchangeably with extension. Including short-term trials in this review is important in part because recent findings are included that were not available in earlier reviews. Maintenance ADM research for youth with MDD is then reviewed. Maintenance for youth who responded to short term trials are divided into: a) extended trials which are continuations of ADM short-term trials, and b) placebo substitution maintenance trials.

The analysis reported herein is the product of an extensive search of the literature to which the many references will attest. The review is based on articles and meta-analyses published primarily since year 2000.

## Short-Term Efficacy Trials

1. ADM short-term trials (usually lasting 8–12 weeks) for youth diagnosed with MDD reveal no meaningful benefit for drugs over placebo in children and a marginal benefit for drugs over placebo in adolescents (7–9).
2. In the five most recent ADM short-term trials (2010–2018) of youth treated for MDD, the median drug response rate was 65% (range: 59%–78%), and the median placebo response rate was 60% (range: 54%–63%) (10–14). The overall 5 percentage point drug-placebo ADM response difference is clearly not clinically meaningful.
  Three of the trials were comparator (drug-drug-placebo) trials (11, 12, 14). Such trials tend to have higher placebo responses due to the reduced risk and reduced expectancy of receiving a placebo (15). The impact of this comparator effect was assessed for youth and found to be present but statistically nonsignificant (15).
3. In the three most recent efficacy trials for youth with MDD (published in 2014 and 2018) that included fluoxetine as a comparator, the placebo rates of response were 60%, 62% and 63% (11, 12, 14). In the only other fluoxetine response data from short-term ADM trials for youth diagnosed with MDD (published in 1997, 2002 and 2004), the placebo response rates ranged from 33% to 37% (16, 17). The comparatively low placebo response levels in the three earlier short-term fluoxetine trials were obviously the major factor that led to the positive trial outcomes. It is not clear why the earlier placebo responses in fluoxetine trials were so low. In a pooled analysis of 23 ADM trials (1972–2007), the average placebo response for youth diagnosed with MDD was 50% (18).
4. In a pooled analysis of 13 efficacy trials (covering years 1997–2006) for youth diagnosed with MDD, average placebo responses were 58% for children and 49% for adolescents (7). Placebo rates were also highest for males, those with fewer episodes of MDD, and for trials in Europe (16).
5. In a meta-analysis of published ADM efficacy trials of **youth** diagnosed with MDD, the average response rates from 1997 to 2010 were 62% for ADM and 52% for placebo (19). In meta-analyses of ADM efficacy trials of ***adults*** diagnosed with MDD, the average responses (2000–2007) were 53% for ADM and 41% for placebo (20). Thus, in ADM trials for MDD, youth have higher response rates than adults to both ADM and to placebo. Based on recent trials (see item #2 above), youth also experience a smaller ADM treatment minus placebo difference.
6. In summary, evidence supporting ADM efficacy for youth diagnosed with MDD has become increasingly limited. These trials tend now to have higher placebo responses.

## Questions About Maintenance

How much is known about the long-term course of MDD in youth? Does discontinuation of ADM in drug responders create problems? Does including an ADM along with a psycho-social intervention for MDD further improve outcome? To answer these questions, selected published studies covering these and related issues are presented and discussed.

## Extension and Maintenance Clinical Trials

### Proportion of Responders by the End of Extended Trials

At intake before an ADM efficacy trial, most depressed subjects having problematic comorbidities are excluded (21), thus making the selection process unrepresentative. Youth diagnosed with MDD who entered the trial and responded to short-term ADM trials are then encouraged to enter into a 24–36-week extension (open label) trial during which they continue their ADM. Nearly all the ADM responders choose to enter these continuation trials [e.g., (12, 14)]. At the end of the extended trial, a median of 72% (range 51%–100%) of these youth maintain their response (12, 14, 22–30).

Follow-up studies show a similar degree of improvement following brief psychological treatment (with or without an ADM) for an MDD episode (31–34). Likewise, a review of the natural course of MDD in youth by Birmaher et al. (35) reported that approximately 90% of youth who experienced an MDD episode remit within 1.5–2 years after its onset.

Essentially, the results of extended ADM trials reveal that most highly selected youth diagnosed with MDD who achieved a response during short-term efficacy trials continue to do relatively well symptomatically during a 6- to 8-month medication extension trial. This evidence is of interest, but it does not support that a response during continued ADM treatment is associated with a better outcome compared to alternative interventions or the natural course of the disorder.

### Relapse During Extension Trials

In many 24–36-week ADM extension trials, relapse/recurrence rates were not reported [e.g. (14, 27, 36)]. When recorded, relapse rates were judged to occur when depression symptom scores substantially rose for at least two weeks. In six trials, the median relapse/recurrence rate was 34% (range 25%–42%) (22, 34, 37–40). Naturalistic follow up studies on MDD in youth are few and their findings on relapse depend heavily on the number of prior depressive episodes. In such studies, recurrence rates in 1–3 years were estimated to be ∼40% (41–43).

### Dropout Rates During Extension Trials

Dropout during ADM clinical trials often includes relapses since many who relapse leave the study. In 10 extended 24–36-week ADM trials, the median trial dropout rate was 45% (range: 30% to 77%) (22, 24, 26, 27, 30, 39, 44–47). Most dropouts in maintenance studies occur for administrative or for unknown reasons (14, 22, 24, 46) and relatively few dropouts are due to side effects (48). Attrition rates are important since they can create serious statistical problems in assessing outcomes. Naudet et al. (49; p. 223) for example, reported that dropout rates as low as 20% in controlled trials “…can cause biased estimates of the treatment effect and restrict the scope for generalizing results.”

### Controlled Extension Trials

Extension/continuation ADM trials were generally not controlled. Trials that were controlled include: a) those comparing cognitive behavior therapy (CBT) with (and without) ADM [e.g., (12, 22, 37, 39)]; and b) four placebo substitution (drug discontinuation) maintenance trials comparing the outcome of drug treatment to placebo in short-term trial responders (30, 40, 44, 50).

### ADM vs. CBT Plus ADM Comparison Trials

Eight trial extensions compared ADM to CBT plus ADM. The results are mixed, but in most instances, they showed a small advantage for the treatment combination (22, 25, 28, 29, 32, 37–39). Trials comparing one ADM to another ADM produced no significant drug response differences (12, 14, 39).

### Placebo Substitution Maintenance Trials

Placebo substitution trials begin with carefully selected subjects who experienced a response during short-term ADM trials. The responders are then encouraged to enter into a double-blind, placebo-controlled maintenance trial. In these trials, over 50% of those assigned to placebo experience a relapse within 8 weeks, whereas youth assigned to continue their ADM experience a far lower rate (∼15%) of relapse during that period (37, 40, 50).

In three of the four placebo substitution/drug discontinuation trials that compared ADM to placebo, relapse rates were 2 or more times greater for those assigned to placebo than for those assigned to ADM (40, 44, 50). In a fourth such trial, the differences were notable, but non-significant (30).

Maintenance placebo-substitution trials have been criticized on various grounds. These include a selection bias that excludes ADM efficacy trial subjects who are likely to be non-responders, apprehension about a possible switch to placebo, and the ∼35% risk of withdrawal—initially characterized by dizziness and agitation—which can follow the abrupt cessation of extended ADM treatment (15, 51–54).

Apprehension about being switched from an ADM to placebo after a drug response is more likely for adults than for youth (15). Nonetheless, in one study, 14% of young ADM trial responders refused to enter the double-blind placebo-substitution trial largely due to fear of a sudden switch off their ADM to placebo (50). Only about 65% of ADM efficacy responders enter discontinuation trials (40, 44, 50), whereas over 90% of ADM responders enter extended trials (12, 14). Even those assigned to remain on their ADM do less well in these ‘relapse prevention’ trials than those in extension trials (12, 40, 50).

## Broader issues

### Vulnerability to Relapse During a Trial

Youth diagnosed with MDD in clinical ADM trials who are relatively more prone to relapse are female, have a chronic course, report suicidality, and have comorbidities such as anxiety, substance abuse, and behavior problems (33, 39, 55). These patients tend not to be selected for ADM trials (21). Furthermore, selecting enriched samples for the trial reduces generalizability of the findings (56).

### Bias in ADM Efficacy Trials Including Youth Diagnosed With MDD

In a Cochrane review of antidepressant treatment for depressive disorders in youth, the authors found much to criticize: “We judged none of these trials (n = 19) to be at low risk of bias, with limited information about many aspects of risk of bias, high dropout rates and issues regarding measurement instruments and the clinical usefulness of outcomes, which were often variously defined across trials.” (56: p.1). An extreme example of data distortion is the erroneous publication of ADM study 329 (57), which necessitated a total corrective data reanalysis (58).

### Increasing Rates of Placebo Responses in Recent Decades

In adult ADM placebo-controlled trials since the 1980s, rates of placebo response have steadily increased (59). In a meta-analysis of ADM for adults with MDD, the difference between ADM and placebo averaged 21 percentage points between 1983 and 1997, but only 13 percentage points between 1998 and 2010 (60). The narrowing of the ADM-placebo gap was clearly due to an increase in the placebo response rate during more recent trials—which thereby reduced positive trial findings (15, 18). This placebo increase was possibly due to the selection of subjects with milder degrees of MDD in recent trials (54).

### Absence of Extension Trials With Placebo

There has been a reluctance on the part of short-term trial investigators to maintain placebo responders on a placebo upon entering extension trials. Instead, investigators have switched placebo responders to an ADM at that transition point (12, 14).

### The Need for More Post-Marketing Research

The NIMH has fortunately sponsored naturalistic long-term follow up studies of numerous funded clinical trials for youth (e.g., TADS). But in ADM research for youth, the NIMH has rarely sponsored post-marketing cohort studies retrospectively assessing outcome using large available administrative datasets. Likewise, they have not funded prospectively-assessed cohort studies using electronic medical records from major academic centers. Furthermore, they have not sponsored long term trials that compare ADM with active placebos and with other comparator medications (61).

In research studies, the natural course of a MDD episode in youth varied from a median duration of 3 months to a mean duration of 8 months (35, 62). It has not been shown that a remission in the natural course of MDD measurably differs in duration from that following an ADM short-term trial (if it is adjusted for baseline levels). Indeed, many of the dropouts during trial extension might simply be part of a “natural course” symptomatic remission.

### Related Concerns

Even though it is somewhat beyond the scope of this review, there are a great many other legitimate concerns relating to ADM maintenance for youth diagnosed with MDD. Briefly, these include: a) the weak reliability of the MDD diagnosis in youth (63); b) the limited generalization of trial data for clinical practice; c) the misleading depiction of short-term trial findings as the primary evidence base for psychiatric treatment (64); d) the fact that most youth in communities treated for depression with ADMs have subthreshold levels of depression (65); e) the common finding that patients with subthreshold depressive symptoms are less likely to benefit from antidepressants (66);–although they are equally likely to experience harms; f) the consistent finding that NIMH sponsored short-term clinical trials for youth show no evidence that trial assignment predicted outcome at follow up [e.g., (33, 67)]; g) the fact that social/interpersonal/situational/economic factors have a distinctly greater impact on MDD outcome than ADM treatment—as shown in the Sequenced Treatment Alternatives to Relieve Depression (STAR*D) ADM trial for adults (68, 69); and h) the evidence that increases in community ADM treatment have not been shown to reduce the population prevalence of MDD (70–72).

## Limitations of This Review

This research did not review tricyclic ADM efficacy and extended clinical trials before 1997, antidepressant adverse drug events, ADM maintenance treatment for OCD, trial vs. community rates of ADM non-adherence, or ADM outcome findings comparing NIMH vs. industry sponsorship.

## Concluding Remarks

If one excludes the placebo substitution maintenance trials because of their serious methodological flaws (described above), then available research support for ADM treatment during maintenance for youth diagnosed with MDD loses its foundation. Furthermore, the fairly frequent occurrence of MDD recurrent episodes during prolonged ADM treatment raises serious questions about the appropriateness of the term “relapse prevention” for ADM maintenance. Continued mental health intervention for youth diagnosed with MDD is usually useful and often necessary (73). Including ADM (apart from its “placebo impact”) during continued psychosocial treatment of youth with MDD requires far better evidence of benefit than is presently available.

## Author Contributions

DS wrote the mini review and JZ made substantive changes and additions.

## Conflict of Interest

The authors declare that the research was conducted in the absence of any commercial or financial relationships that could be construed as a potential conflict of interest.
